# Spatial Heterogeneity in Human Activities Favors the Persistence of Wolves in Agroecosystems

**DOI:** 10.1371/journal.pone.0108080

**Published:** 2014-09-24

**Authors:** Mohsen Ahmadi, José Vicente López-Bao, Mohammad Kaboli

**Affiliations:** 1 Department of Environmental Sciences, Faculty of Natural Resources, University of Tehran, Karaj, Iran; 2 Research Unit of Biodiversity (UO/CSIC/PA), Oviedo University, Mieres, Spain; 3 Grimsö Wildlife Research Station, Dep. of Ecology, Swedish University of Agricultural Sciences (SLU), Riddarhyttan, Sweden; Instituto de Pesquisas Ecológicas, Brazil

## Abstract

As human populations expand, there is increasing demand and pressure for land. Under this scenario, behavioural flexibility and adaptation become important processes leading to the persistence of large carnivores in human-dominated landscapes such as agroecosystems. A growing interest has recently emerged on the outcome of the coexistence between wolves and humans in these systems. It has been suggested that spatial heterogeneity in human activities would be a major environmental factor modulating vulnerability and persistence of this contentious species in agroecosystems. Here, we combined information from 35 den sites detected between 2011 and 2012 in agroecosystems of western Iran (Hamedan province), a set of environmental variables measured at landscape and fine spatial scales, and generalized linear models to identify patterns of den site selection by wolves in a highly-modified agroecosystem. On a landscape level, wolves selected a mixture of rangelands with scattered dry-farms on hillsides (showing a low human use) to locate their dens, avoiding areas with high densities of settlements and primary roads. On a fine spatial scale, wolves primarily excavated dens into the sides of elevated steep-slope hills with availability of water bodies in the vicinity of den sites, and wolves were relegated to dig in places with coarse-soil particles. Our results suggest that vulnerability of wolves in human-dominated landscapes could be compensated by the existence of spatial heterogeneity in human activities. Such heterogeneity would favor wolf persistence in agroecosystems favoring a land sharing model of coexistence between wolves and people.

## Introduction

As human populations expand, there is increasing demand and pressure for land (characterized by an increment and expansion in settlements, habitat transformation and extension of agricultural lands, and industrial development) and, consequently, different impacts on wildlife are expected. Under this scenario, behavioural flexibility and adaptation are important processes leading to the persistence of viable animal populations in human-dominated landscapes, including urban environments (e.g. mammalian carnivores [1], [2]). For species like large carnivores, with remarkable large spatial requirements, low reproductive rates or low densities [3], as well as a high potential for conflict (e.g. livestock attacks [4], [5]), such behavioural processes are key elements determining their persistence in human-dominated landscapes. In fact, the capability of these species to persist in this scenario, and its behavioural, demographic and ecological consequences, have attracted a great attention in recent times [2], [6], [7], [8].

Existing evidence shows how wolves (*Canis lupus*) are able to persist in contrasting human-dominated landscapes [7], [9], [10], [11], [12] as soon as legislation is favourable and human pressure is low [13], and minimum food and refuge requirements are fulfilled [3]. Several mechanisms are behind this ability such as the spatio-temporal segregation between wolves and human activities [9], [14], their capacity to use different human-related sources of food [15], [16] or other behavioural adaptations such as den shifting [17]. All this information suggest that wolves are highly capable to persist in humanized landscapes by perceiving mortality risk associated with humans, adjusting, for instance, the use of the space at different scales over time accordingly [7], [17], [18] (see [2] for an example with the red wolf). Thus, the spatial and temporal heterogeneity in human activities would emerge as a major environmental factor modulating vulnerability and persistence of wolves in human-dominated landscapes, resulting in wolf persistence even in areas completely transformed by humans [2], [7], [18], [19].

In agroecosystems, ecological systems modified by human beings to produce food, fibre or other agricultural products [20], such heterogeneity in human activities may provide wolves with places of low human use where they can go unnoticed and, more importantly, can reproduce. Although the impact of humans on wolf persistence has been inferred using different surrogates such as human population density, infrastructures, level of transformation of the landscape or the spatial distribution of activities [7], [21], [22], how these human-related factors interact with the persistence of wolves in agroecosystems remains poorly understood. However, this knowledge becoming particularly important owing to the recent expansion of wolf populations and human activities, particularly agriculture [8], [23], being crucial to adopt a balanced landscape planning ensuring both, human needs and wolf persistence [22]. Moreover, understanding the abilities of wolves to persist in each particular local context is a pressing need to reach a context-dependent conservation and management approach in agroecosystems, since heterogeneity is the norm across human-dominated landscapes [24].

Reproductive success is a cornerstone for the persistence of any species. For large carnivores, reproductive success is highly influenced by humans [3]. Because the highest mortality rate of wolves occur in the first months of their life [25], [26], selection of the place where to locate the den site is crucial for wolves, being particularly important in human-dominated landscapes [17], [27]. Available information suggests that, in agroecosystems, exposure risk to humans will exert the strongest effect on den site selection, with wolves aiming to minimize such risks. As a result, even in completely transformed landscapes wolves may place their den sites in areas where human activities are low [2], [18], [19]. In addition, the strength of human activities driving the selection of den sites by wolves in these systems may force other natural components of this selection process to the background. For example, in many areas wolves select for sites where they can dig easily [9], [28], but in agroecosystems, where intensive cultivation practices are preferable on good soil conditions, wolves may be forced to dig in low-quality sites in terms of soil conditions.

In this study, we aimed to identify patterns of den site selection by wolves in agroecosystems of western Iran (Hamedan province), and provide insights into the behavioural response of wolves to the spatial heterogeneity in human activities. Since large-scale approaches may disregard fine-scale patterns affecting different components of the selection processes we were interested, we evaluated the requirements of denning wolves at large (den area) and fine (den site) spatial scales. In particular, we hypothesized that wolves are able to assess the type and intensity of human activities over a wide geographic range selecting den areas with low human use, minimizing the risk of mortality. Thus, on a landscape level, we first expect that wolves will avoid areas with high densities of infrastructures and humans and, second, we also predict that, in absence of natural dense vegetated areas in this agroecosystem acting as refuge and where to locate the den sites, wolves will select farmlands with the lowest intensity of human activity. On a fine scale, we expect that although wolves will select for den sites fulfilling previous known environmental requirements for the species (e.g. water availability, refuge, human inaccessibility, [9], [28], [29], [30], [31]), the strength of humans activities influencing den site selection in agroecosystems may push some components of the selection process into the background as a response to minimize the risk of exposure to humans.

## Materials and Methods

### Study area

Despite extensive studies on wolf distribution, biology, ecology and behaviour (see review in [7], [11], [32], [33], [34]) and conflict with humans (e.g. [4], [5]) in Europe, North America or India, wolves are less studied in the Middle East. However, conflicts between wolves and humans are considerable in anthropogenic landscapes of Iran, affecting the attitudes of rural communities and the conservation status of the species [35], [36], [37].

**Figure 1:**
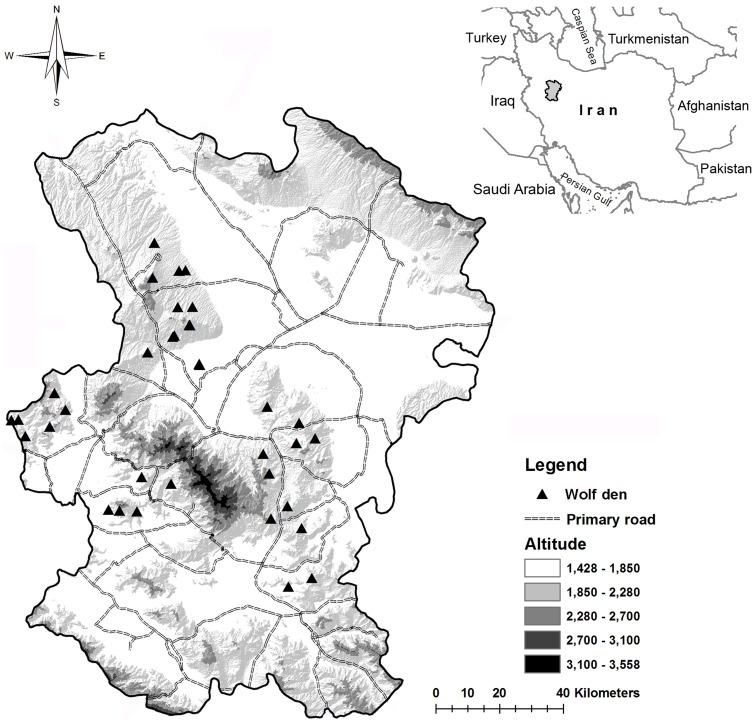
Distribution of gray wolf dens detected between 2011 and 2012 in Hamedan province, Iran. Wolf dens were overviewed in a context of topography and main roads in Hamedan province, Iran.

**Figure 2:**
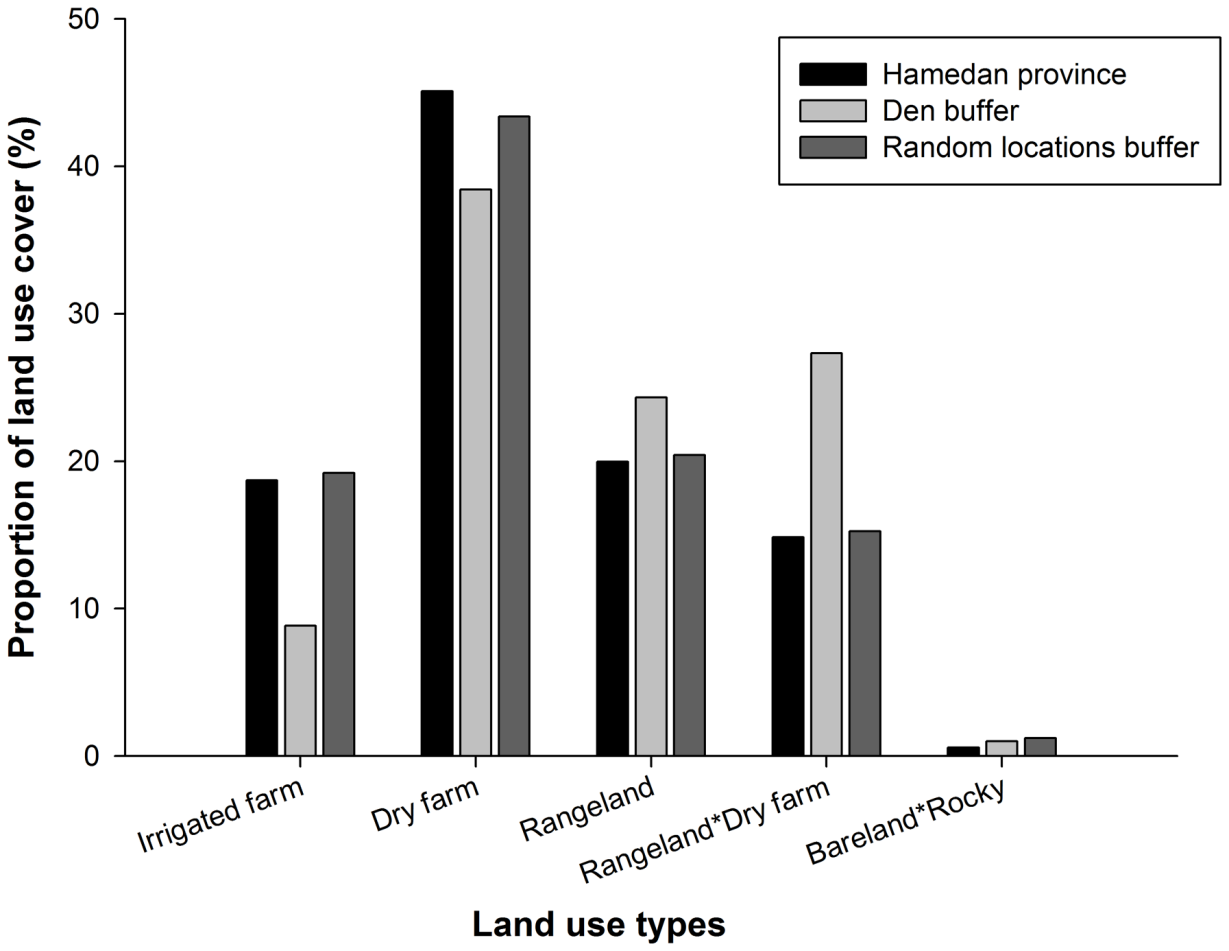
Proportion of land use/land cover categories used in this study. Proportion of each cover type was calculated within 2 km circular buffers around wolf den sites (den areas), random locations (random areas) and proportion of the whole study area (Hamedan province).

This study was carried out in Hamedan province, a human-dominated landscape located in western Iran (88 inhabitants/km^2^; Fig. 1) [38] and covering an area of 19,546 km^2^ (47°34′ – 49°36′ E and 35°25′ – 35°15′ N; Fig. 1). The region has a cold semi-arid climate with an average annual precipitation of 325 mm and a mean annual temperature of 11°C. The landscape in Hamedan province is severely transformed because traditionally rural community has been mostly engaged in agriculture and livestock rearing and husbandry. Consequently, agricultural lands dominate this semi-arid landscape ([39], Fig. 2; Figure S1). The very few (2% of the whole province), and small in size, patches of natural vegetation - composed by shrub species such as *Astragalus* spp. and *Bromus* spp. and with scattered trees such as Persian oak (*Quercus brantii*), Dogwood (*Cornus australis*) or Cherry plum (*Prunus divaricata*) [40] - are distributed within a heterogeneous agricultural matrix composed by intensive irrigated potato and corn farms, dry-farms (cereals) and rangelands – which are used for extensive grazing - with scattered dry-farms (Fig. 2, Figure S2). Landscape transformation has been dramatic in this area in recent times resulting in an increase of agriculture lands from 20,468 ha to 550,264 ha during the past 30 years [39]. Consequently, rangelands covered by perennial bushes and grasses decreased from 539,697 ha to 164,679 ha [39]. The expansion of agriculture lands have significantly reduced the amount of natural refuge for wolves in this open landscape and, at the same time, have also reduced wild prey populations [39], thus increasing human-wolf encounters and associated conflicts [37].

Small variations in topographic attributes - altitude and slope - in this plateau (most of the area ranges between 1,500 and 2,000 m.a.s.l and slope changes between 0 to 41 degrees) strongly determine the use of the landscape by local people. Thus, while flat areas (slope <10 degrees) are the most preferred landscape for settlements, development and human activities (84.5% of the study area), rugged landscapes (slope> 10 degrees) only encompass 15.5% of the whole landscape and is mainly used as rangelands and, sometimes, dry-farms. As a result, human activities are heterogeneously distributed across different types of farmlands. Based on cultivation and livestock practices and land use, intensity of human activities differ across farmlands as follow: irrigated farms> dry farms> rangeland with scattered farms> rangelands. For example, in irrigated farmlands (e.g. potatoes, corn), the use of heavy equipment and mechanized cultivation is quite common and these type of crops requires a continuous human presence during many months of the year, including the peak of reproductive activity of denning wolves. On the other hand, cultivation strategies of other types of farms such as dry-farms require human presence only in two specific periods, plant and harvest, resulting in low human presence especially during denning activities and rearing of immature pups.

### Data collection

We used information from 35 den sites detected between 2011 and 2012 (5 den sites in 2011 and 30 in 2012; all den sites were different). Wolf dens were located using information from local sources in the rural areas, especially observations from sheepherders and game guards of the Department of Environment of Hamedan province, as well as field patrols conducted by motorcycle in those areas where we expected to find wolf dens according to previous local knowledge in the area. Since all issues subject to wildlife care and animal welfare regulations is handled by Department of Environment (DOE) In Iran, as well as the study was in collaborated with Hamedan Provincial Bureau of Department of Environment (43106/140), all our fieldwork procedures was adhered to the animal welfare regulations. Our data sampling was carried on after confirming that wolf packs left their dens. Our field survey did not involve chasing the wolves to locate their dens. We also did not destroy or damage wolf dens. Since the breeding season is the most sensitive period for wolves [32], once a potential den site was found, we approached to the site when the pups were out of the den (between May and June) to confirm wolf reproduction. After dens were located and absence of wolves and pups was ensured, we took the location of the den sites with a GPS unit and measured the fine-scale variables we were interested (see below).

Data sampling and measurement of environmental variables were performed in two different spatial scales and using different protocols: i) den area (12.5 km^2^; landscape scale), where environmental variables were measured by using GIS; and ii) den site (0.01 km^2^; fine scale), where variables were measured *in situ*. On a landscape level, we estimated the spatial heterogeneity in human activities around den areas using a 2 km circular buffer centered on the den sites. The lack of information on wolf territory size in the study area confined us to consider a 2 km buffer size based on literature review [28], [41], which well-describes landscape characterization of den areas [42]. For non-den areas we randomly selected 100 non-overlapping circular plots with the same radius excluding the largest cities and areas with an altitude of higher than 3,000 m.a.s.l. Because of the extensive movements of wolves, the distance between random and observed (den sites) points was controlled not to fall below 15 km. This conservative distance was selected based on published empirical values of the nearest neighbor distance for active breeding dens of wolves [33], [42].

The spatial heterogeneity in human activities was inferred using three different surrogates (Table 1). First, we calculated the proportion of each land use type on a landscape-level (2 km circular buffer) using the Iranian Forests, Range and Watershed Management Organization National land use/land cover map [43]. We focused on four categories of land use representing the above-mentioned gradient in the intensity of human activities (irrigated farms> dry-farms> rangeland with scattered farms> rangelands). We excluded bare lands and rocks areas due to its anecdotic representation in the area (Fig. 2). Second, we used density of settlements and length of roads as a surrogate of human intrusion and risk of mortality in the landscape. These factors are well-known affecting wolf habitat selection in general [7], [11], [34], and den site selection in particular [9], [31]. Density of settlements and length of roads were calculated from topographic military maps of Iran with a 1∶25,000 scale. Because of the different response of wolves to road networks with varied level of human activity [11], [33], we classified road networks into two categories: primary roads, including national primary roads and highways with bound> 45 m, and secondary roads, including regional and district roads with bound <30 m.

**Table 1:** Mean (SE) values of variables measured at the level of the den area, in 2 km circular buffers with and without wolf dens in Hamedan province, Iran.

Variables (unit)	Abbreviation	Den areas	Random areas	P-value
Dry farms (%)	Dry	39.8 (3.5)	40.3 (3.3)	0.770
Irrigated farms (%)	Irgt	8.5 (2.4)	22.3 (3.2)	0.050
Rangeland (%)	Rng	22.1 (4.4)	19.5 (2.9)	0.259
Rangeland with scattered farms (%)	Rng_Dry	28.3 (4.5)	13.6 (2.3)	0.001
Bareland and Rocks (%)	Bare	0.01 (0.01)	1.3 (0.8)	0.912
Altitude (m)	Alt	2116.0 (20.4)	1999.4 (25.8)	0.000
Roughness (m)	Rough	55.9 (5.1)	49.7 (5.4)	0.022
Length of primary roads (km)	Prim	0.4 (0.2)	1.6 (0.3)	0.010
Length of secondary roads (km)	Scond	2.5 (0.4)	2.2 (0.3)	0.190
Density of settlements (%)	Setl	0.0027 (0.0005)	0.0130 (0.0026)	0.034

Comparisons between den areas and random areas were done by Mann–Whitney U-tests.

Third, using the Shuttle Radar Topography Mission elevation model with 100 m resolution, we compiled mean altitude and roughness as the main factors describing the topographic context of each area which is expected to be correlated with human activities as mentioned above (human activities decrease with the increment in altitude and roughness; [7], [44]). For each den area, we then calculated the mean altitude (m) by averaging altitudes of all raster cells included in this area, and roughness (m) was estimated as the standard deviation of the altitudes of all the 100 m raster cells included in each den area. Both measures reflect different types of human use; i.e. flat areas are preferred for intensive agriculture whereas rough surfaces are more inappropriate to use farm machinery being used for extensive livestock practices and dry-farms. Vegetation types providing structural protection to wolves, such as scrubs or forests, are often selected as refuge [7], [34]. But semi-arid agroecosystems of Iran, as well as other open semi-arid landscapes within the wolf's range [18], lacks such suitable cover types to provide concealment for wolves. Hence roughness of terrain that is taken into account in this study could be a representative of concealment for wolf movements [7], [35].

On a fine-scale (100 m radius), we measured thirteen variables related to the vulnerability of wolves (vegetation types and slope as surrogates of refuge, human activity – existence of farmlands -), ease to dig (soil/petrology; soil type and rock density can affect den site selection by wolves [17], [47]), water availability, which may be a determinant factor to locate the den [28], [30], particularly in arid environments, along with solar insulation. These variables were chosen based on their suggested importance for wolf den site selection in other temperate study areas [9], [28], [29], [30], [31]. Excepting for solar insulation, all fine-scale variables were measured in five 20 × 20 m plots, one centred at the den opening and the other four plots 50 m far from the den opening in the cardinal directions [29]. We averaged all variables measured in the five plots, excluding water availability and existence of farmlands that were categorized as a binary factor, to get a general overview of the surroundings of the den and to provide a realistic distribution of the selected variables in den sites. We used hillshade as a surrogate of solar insulation [45]. Hillshade was calculated by combining slope and aspect in the den site and using ArcGIS 9.3 [46]. Hillshade values represent the average amount of shade per year received at any point. Thus, warmer slopes (facing southwest) will receive the greatest hillshade values, whereas cooler northeastern slopes will correspond to the lowest hillshade values. Due to the lack of information on accurate home range size of wolves in the study area, we conservatively selected absence plots to measure the same variables for the fine-scale analysis 1 km away from the den in a random direction (i.e. random points; equal number of points per known den sites), where we were ensured of the absence of wolf dens [29], [30]. Out of the 35 den sites detected, fine-scale data sampling was carried out in 32 dens (3 den sites were destroyed before we could measure fine scale variables).

### Statistical Analyses

In a first step, we carried out univariate analyses (Mann–Whitney U-tests) testing for significant differences between wolf den areas/sites and non-wolf den areas/sites for all the explanatory variables, excepting for the proportion of den sites with water bodies and farmlands within 100 m radius, where Z-proportions tests were used (Table 1, Table 2). At fine scale, we also used principal component analysis (PCA) to extract orthogonal multivariate axes on fine-scale soil-petrologic variables (Table 2). PCs obtained were used to identify the combination of inter-correlated petrologic measurements into organized components that best separate used/unused wolf den sites. We extracted the first two components (PC1soil and PC2soil, Table 2) which explained 73% of soil characteristic variance in measured plots and used them as den site descriptive variables for soil conditions. PC1soil was related to coarse particles of soil and rocks and PC2soil indicated fine soil particles (i.e. optimum areas for cultivation; Table 2).

**Table 2:** Mean (SE) values of fine-scale variables measured in sample plots with and without wolf dens in Hamedan province, Iran.

Variables	Description	Den sites	Random points	P-value
Slope	Measured by a clinometers	15.4 (6)	9 (3.3)	0.000
Hillshade	Measured by a combination of slope and aspect	175.5 (2.11)	177.8 (3.24)	0.234
Herbaceous	Vegetation height less than 25 cm (percentage)	53.3 (20.6)	70.9 (21.4)	0.008
Shrub	Vegetation height between 25 to 200 cm, (percentage)	43.6 (18.3)	25.1 (20.4)	0.003
Tree	Vegetation height above than 200 cm, (percentage)	3.1 (5.3)	3.9 (5.6)	0.406
Soil/Petrology (proportion)	Sable: Particles of clay and sand	35.8 (11.5)	60.6 (7.5)	
	Mm: Soil particle ≤ 1cm	33.8 (7.8)	28.7 (5.3)	
	Cm: Pebbles with size of ≤ 10 cm	15.9 (6.3)	8.4 (3.2)	
	Dc.m: Pebbles with size of ≤ 1 m	9.5 (6.4)	3.2 (2.3)	
	M: Rock with size of ≤ 10 m	5 (6.5)	1.4 (1.5)	
	Dca.m: Rocky materials with size of> 10 m	1.7 (3.5)	0.1 (0.7)	
	PC1 soil: first component of PCA analysis preformed onSoil/Petrology - coarse soil particles -			0.006
	PC2 soil: second component of PCA analysis preformed on Soil/Petrology -fine soil particles -			0.004
Water availability	Proportion of sites with water bodies within 100 m radius	0.75	0.31	0.001
Farm	Proportion of sites with farmlands within 100 m radius	0.56	0.72	0.283

Comparisons between den sites and random sites were done by Mann–Whitney U-tests excepting for the proportion of presence of water bodies and farmlands within a 100m radius, which were evaluated using Z-proportions tests.

For both spatial scales, we built separate Generalized Linear Models (GLMs) with binomial error distribution and logit link to assess the influence of human activities on den site selection patterns by wolves in this semi-arid agroecosystem. For each spatial scale, Pearson correlation coefficients were used to test for multicollinearity among predictors, but no significant correlation between any pair of explanatory variables was detected. At the landscape scale, because of the inherent relationship between topographic contexts with land use, we first examined the possible interactions between elevation and roughness against land use types and length of primary and secondary roads (Table S1), and significant interactions were included in the full model. To do this, we generated a set of additional GLMs containing the pairwise interaction of each land use and type of roads with elevation and roughness (Table S1). We then used the “*anova*” function of the “car” package for R [48] to calculate Likelihood-Ratio χ^2^ and Wald χ^2^ in order to evaluate the significance level of each interaction. Akaike's Information Criterion corrected for small sample sizes (AICc) [49] was used for model selection and multi-model inference. For each spatial scale, we selected models with ΔAICc <2, and we calculated Akaike weights (AICc *wi*) [49]. Moreover, for each predictor selected in the set of models with ΔAICc <2, we calculated its estimated importance (or relative evidence weight), computed as the sum of the relative evidence weights of all models in which the variable appears, as well as model-averaged estimates and their unconditional standard errors (SE). Using this approach we reduced model selection bias effects on regression coefficient estimates in all selected subsets [49]. Finally, to verify how well the selected models described our dataset, we performed a Goodness-of-fit test using Hosmer-Lemeshow (HL) procedure [50]. The Area Under the Curve (AUC) of ROC was also calculated as a measure of discrimination capacity of selected candidate models. All analyses were carried out in R version 3.0.1 [51].

## Results

### Breeding in agroecosystems

Den areas were located in agricultural matrix with a significantly less proportion of irrigated farms (Mann–Whitney U-test, *P* <0.05; Table 1) and a higher proportion of mosaics of rangelands with scattered dry-farms than random areas (Mann–Whitney U-test, *P* <0.001; Table 1). We did not find significant differences between den and random areas for the rest of land uses (Table 1). Wolves tended to select elevated and rough areas (where intensive agricultural practices, such as irrigated farms, are less probable; altitude: Mann–Whitney U-test, *P* <0.0001; roughness: Mann–Whitney U-test, *P* = 0.022; Table 1, Table S1). Finally, as predicted, wolves also avoided areas with abundant primary roads and density of settlements (primary roads: Mann–Whitney U-test, *P* = 0.017; settlements: Mann–Whitney U-test, *P* = 0.016; Table 1). However, location of den sites was not influenced by the development of the network of secondary roads in the den area (Table 1).

We found a significant interaction between irrigated farms and roughness (χ^2^ = 6.147, *P* = 0.013; Table S1), and between altitude and secondary roads (χ^2^ = 3.967, *P* = 0.043; Table S1). Hence these two interactions were included in the set of predictors for the landscape scale models. Seven candidate models showed ΔAICc <2 (Table 3), with the best model including rangelands with scattered dry-farms, altitude, roughness, human settlements, primary roads and the interaction between irrigated farms and roughness (Table 3). The probability of a given area being selected as a den area by wolves in this semi-arid agroecosystem raised with an increase in the proportion of rangelands with scattered dry-farms, located at high altitudes and with low human presence (negative estimation for length of primary roads and density of human settlements; Table 4). Model-averaged coefficient estimates indicated that rangelands with scattered dry farms, altitude, roughness, primary roads and human settlements were the most important predictors determining the probability of a given area being selected as a den area by wolves (Table 4). AUC of ROC curve showed good discrimination capacity of selected candidate models and, we did not find evidence of lack of fit in the different models (HL tests, Table S2).

**Table 3:** Selected candidate Generalized Linear Models explaining gray wolf den area selection patterns in Hamedan province, Iran, at the landscape level.

Model	AICc	ΔAICc	AIC *w_i_*
Rng_Dry + Alt + Rough + Setl + Prim + (Irgt × Roug)	125.12	0.02	0.18
Rng_Dry + Alt + Rough + Setl + Prim	125.28	0.18	0.16
Rng_Dry + Alt + Rough + Setl + Prim + (Irgt × Roug) + (Alt × Scond)	126	0.90	0.11
Rng_Dry + Alt + Rough + Setl + Prim + (Alt × Scond) + Dry	126.21	1.11	0.10
Rng_Dry + Alt + Rough + Prim + (Irgt × Roug) + (Alt × Scond)	126.45	1.35	0.09
Rng_Dry + Alt + Rough + Setl + Prim + (Irgt × Roug) + Rng	126.81	1.71	0.07
Rng_Dry + Alt + Rough + Setl + Prim + (Irgt × Roug) + Irgt	127.06	1.96	0.06

Models were ranked according to AICc, and only models with ΔAICc <2 are shown for simplicity. For variables description see Table 1.

**Table 4:** Relative importance (W+), model-averaged coefficient estimates (Estimate), and unconditional standard errors (SE) for the predictors included in the selected candidate models determining the probability of a given area being selected as a den area by wolves in Hamedan province, Iran (models with ΔAICc <2).

Variable	W+	Estimate	SE
Intercept		−4.71	3.71
Rng_Dry	1	0.02	0.01
Alt	1	0.002	0.001
Roug	1	0.02	0.01
Setl	1	−0.01	0.05
Prim	1	−0.003	0.002
Irgt × Roug	0.95	0.001	0.001
Alt × Scond	0.83	0.0004	0.002
Irgt	0.35	−0.001	0.002
Rng	0.27	0.02	0.01
Dry	0.10	−0.02	0.01

For variables description see Table 1.

### Fine-scale den site selection patterns in agroecosystems

Wolves were prone to excavate dens in rough hillsides with moderate shrub cover (Mann–Whitney U-test, *P* <0.05; Table 2). At fine-scale, the strongest significant difference between occupied and unoccupied sites was slope (15.4±6.0 vs. 9.0±3.3; Mann–Whitney U-test, *P* <0.0001; Table 2). In addition, den sites were characterized by significantly lower percentage of open areas (dominated by herbaceous) as well as higher shrub cover (43.6±18.3 vs. 25.1±20.4; Mann–Whitney U-test, *P* = 0.003; Table 2). Water availability was significantly higher in den sites (Z = 3.276; *P* <0.001; Table 2) and wolves tended to locate them in areas with a high proportion of coarse soil particles (Mann–Whitney U-test, *P* = 0.0004; Table 2). As expected, because the study area was dominated by humans, the presence of farm-lands did not differ between occupied/unoccupied sites at fine scale (Z = 1.073; *P* = 0.283; Table 2). Also, the difference of the amount of shade received at wolf den and random points was not significant (Table 2).

For den sites, eight candidate models showed ΔAICc <2 (Table 5) and the best model included slope, soil/petrologic terms (PC2soil; fine soil particles) and water availability. These three variables were the most important fine-scale predictors of den site selection by wolves based on their relative importance (Table 6). Averaging the coefficient estimates of the selected candidate models revealed that wolves selected for sites with availability of water bodies, placed in stepper hills and with coarse soil particles (Table 6). Based on AUC, we found a very good discrimination capacity for the selected candidate models ranging from 0.915 to 0.933, and no evidence of lack of fit was detected (HL tests, Table S2).

**Table 5:** Selected candidate Generalized Linear Models explaining gray wolf den site selection patterns in Hamedan province, Iran, at the fine spatial scale.

Model	AICc	ΔAICc	AICc *w_i_*
Slope + PC2 soil + Water	55.31	0	0.17
Slope + PC2 soil + Water + Hillshade	55.88	0.56	0.13
Slope + PC2 soil + Water + Herbaceous + Shrub	56.33	1.02	0.10
Slope + PC2 soil + Water + Hillshade + PC1 soil	56.36	1.05	0.10
Slope + PC2 soil + Water + PC1 soil	56.45	1.14	0.10
Slope + PC2 soil + Water + Hillshade + Shrub	56.58	1.27	0.09
Slope + PC2 soil + Water + Shrub	56.66	1.35	0.09
Slope + Water + Herbaceous + Shrub	57.25	1. 94	0.06

Models were ranked according to AICc, and only models with ΔAICc <2 are shown for simplicity. For variables description see Table 2.

**Table 6:** Relative importance (W+), model-averaged coefficient estimates (Estimate), and unconditional standard errors (SE) for the predictors included in the selected candidate models determining the probability of a given site being selected as a den site by wolves in Hamedan province, Iran (models with ΔAICc<2).

Variable	W+	Estimate	SE
Intercept		−6.68	8.09
Slope	1	0.31	0.09
Water	1	3.10	1.04
PC2 soil	0.94	−0.87	0.45
Hillshade	0.48	−0.03	0.02
Shrub	0.39	0.11	0.10
Herbaceous	0.29	0.14	0.09
PC1 soil	0.20	0.29	0.25

For variables description see Table 2.

## Discussion

Humans are the main source of disturbance for large carnivores affecting, for example, the composition and security of their habitats [52]. Wolf distribution and habitat suitability is mainly influenced by human-associated factors [32]. Such human influence can be both direct (i.e. mortality; legal hunting, poaching, road kills) [32], [53] and indirect (behaviour), for example, wild prey depletion or availability of human-related sources of food [15], [16]. However, wolves, as many other large carnivores [2], [6], [8], do not strictly required areas devoid of humans, showing a high ability adapting to multiple used landscapes. This phenomenon is particularly interesting in agroecosystems where virtually all habitats are agricultural and transformed and wild prey can be rare, with wolves usually feeding on livestock, waste or animal carcasses [7], [15], [18], [54].

In agroecosystems, simply avoiding transformed land cover types is impossible, such as the case of western Iran with the almost complete loss of natural habitats (2%) [39]. As a consequence, wolves are relegated to utilize non-natural land cover types while avoiding negative interactions with humans [2], [7], [18], [19]. So, understanding how wolves adjust the use of space in agricultural lands (one of the most widespread habitats worldwide), adapting to human activities, is therefore a critical step to ensure the persistence and conservation of this species in agroecosystems minimizing human-wolf conflicts. This is particularly important since the occurrence of this contentious species in agroecosystems is beyond anecdotic, with several packs occurring, for example, in our study area, as reflected by the number of wolf dens [35] used here [17], [18] (see also [55] for a similar scenario in Spain).

Based on the comparison of human land use between den areas, random areas and the whole study area (Hamedan province) we found that the mixture of rangelands with scattered dry-farms (accounting around 15% of the whole study area; Fig. 2) was preferred by denning wolves, whereas irrigated farms were actively avoided and no patterns were found for extensive and homogeneous dry-farms or rangelands (Fig. 2; Figure S2). The proportion of mixed rangelands with dry-farms was the most predictive variable identifying wolf den areas along with a combined preference for hillsides. Two non-exclusive explanations may be behind of this result. By one hand, dry farming practices requires low levels of human activity, with human presence not overlapping with the most sensitive period for wolves (denning period) because human activity is limited to only the planting and harvesting seasons. On the other hand, rangelands, which can also show a low intensity of human use depending on livestock practices, can also provide wolves with human-related sources of food (e.g. livestock, carrion, waste). Because of the low abundance of wild prey in the area [56] and the use of human-related food sources by wolves in such ecosystems [54], [57], traditional herd roaming in rangelands adjacent to dry-farms by local community may favor food availability (higher density of livestock close to farms), affecting den site selection. On the other hand, this scenario (i.e. the presence of scattered dry-farms) may also increase food availability for scarce wild prey. Further analyses are needed to test these hypotheses.

As we expected, wolf den areas were characterized by lesser density of settlements and primary roads compared with random areas [9], [58]. The lack of difference between den and random areas in the length of secondary roads suggests that having lesser disturbance from main surrogates of human activity (primary roads and settlements; areas with an intense human land use), secondary roads may be a less important limiting factor for den site selection by wolves. In fact, because secondary roads generally show a lower human use, wolves may use these linear infrastructures for ease of travel within their territories [2], [33].

The lack of refuge - considering the well-established link between the concept of refuge and certain vegetation structures providing safe places to wolves such as forests or scrublands [7], [34] - in our study area highlights the importance of rouged terrains with low human use providing good concealment for denning wolves in open areas [7], [58], [59]. Therefore, although wolves selected for den sites located in places with a higher proportion of shrubs compared to random sites in this agroecosystem (Table 2), on a landscape level, vegetation/habitat types becomes a secondary factor for den selection processes, being strongly modulated by the level of human activities.

On a fine spatial scale, our results indicated that wolves primarily excavated dens into the sides of elevated steep-slope hills (Figure S2), selecting sites with steeper slopes, which is consistent with the selection patterns found in other studies (e.g. similar average values for slope, ca. 15 degrees) [29], [60]. The slope in these places will also cause more drainage – in case of torrential rain - than surrounding regions that have gentle slope [29], [31], [47]. Apart from slope, fine soil particles –PC2 soil- (negative selection) and existence of water bodies (positive selection) were the most important variables affecting den site selection patterns. In an unusual pattern, we found that the existence of farmlands did not affect selection patterns by denning wolves [18]. High tendency of local communities to place dry-farms in areas with unsuitable topographic conditions for other cultivation practices may also explains why many dens were located in the vicinity of farmlands. We found a significant difference between den and random sites (that were often located within agricultural lands) in terms of soil variables. Most of the areas with a gentle slope and rich soil (PC2 soil) are used for farming by local people. Accordingly, rangelands adjacent to farms are less usable for agriculture and wolves were forced to den in places with coarse soil particles. Finally, we found that the availability of water bodies in the vicinity of den sites is an important factor for denning wolves. As expected, due to high water requirement of lactating females, den sites were selected relatively close to water sources [28], [29], [30], [60]. In semi-arid landscapes, we predict that the dependency of both, denning wolves and humans, to scarce water bodies may have increased human-wolf conflict locally, being an important limiting factor for the persistence of the species.

Our findings at different spatial scales show how wolves can be tolerant to placing their dens in agricultural lands, which demonstrates their resilience to persist in agroecosystems. As agricultural lands dominated this landscape, wolves selected for den areas with low human use irrespective whether such areas were profoundly transformed or not. In our case, this is possible because small dry-farms adjacent to rangelands require minimum human intervention, consequently having a low impact on habitat security and decreasing the risk of mortality for wolves during the breeding period. Thus, spatial and seasonal heterogeneity in human activities become an important factor explaining the persistence of wolves in agroecosystems [61].

As in other regions of the Middle East, agricultural activities in Hamedan province started more than 5000 years ago [62]. Moreover, contrary to European and North American wolf ranges [63] where wolves were exterminated from huge areas during the 19th and 20th centuries [24], [32], [65], and only began to recolonize some of their former range in recent times [32], such pattern of eradication/re-colonization did not occur in Iran, with wolves persisting in this area continuously over time. Thus, here wolves and human activities have been interacting for a much longer period of time than in other parts of the current and historical wolf range leading to a unique scenario of wolf adaptations to humans.

Effective large carnivore conservation in human-dominated landscape matrix and outside of formally protected areas is of paramount importance in the Anthropocene [64], [65]. Successful conservation strategies requires minimizing conflicts between large carnivores and humans, understanding where and when to establish limits of sharing the landscape with these contentious species. Alternatives range from a focus on fencing large carnivores to allowing them to share the landscape with humans (e.g. [66]). However, this debate also requires determining to what extent large carnivores can tolerate living in human-dominated landscapes considering different spatial and ecological constraints and levels of conflict. Along these lines, our results show how the heterogeneity in human activities emerges as a key factor favoring the persistence of wolves in agroecosystems. Thus, vulnerability of wolves, and other large carnivore species, in human-dominated landscapes could be compensated by the existence of spatial heterogeneity in human activities, favoring a land sharing model of coexistence between large carnivores and people.

However, despite the ability of wolves to persist in agroecosystems, with much of the landscape being devoted to agricultural and livestock activities, human-wolf encounters and conflicts can also increase. As a consequence, because of the high accessibility to wolf dens by people in agroecosystems, lactating wolves and their pups can be very vulnerable to active illegal human persecution [35]. Since wolf core use areas, including den areas, are used by wolf packs more intensively throughout the year and wolves are even prone to use the same den in subsequent years [29], [47], there is a pressing need to adopt efficient measures to mitigate human-wolf conflicts in agroecosystems (e.g. discouraging people from destroying wolf dens, changing human behaviors and livestock practices) in order to keep acceptable levels of tolerance and favoring wolf persistence.

## Supporting Information

Figure S1 **General views of the agroecosystems of Hamedan province, Iran.**
(JPG)

Figure S2 **Fine-scale pictures showing the environment around den sites in rangelands of Hamedan province, Iran.**
(JPG)

Table S1 **Results from Generalized Linear Models testing for significant effects of the pairwise interactions between land use and type of roads with elevation and roughness.**
(DOC)

Table S2 **Results of the assessment of goodness-of-fit and discrimination capacity of selected candidate models explaining the selection of den areas/sites by wolves in Hamedan, Iran.**
(DOC)
