# Supplementary figures and images for: Spatial Heterogeneity in Human Activities Favors the Persistence of Wolves in Agroecosystems
Source: PLoS One. 2014 Sep 24;9(9):e108080. doi: 10.1371/journal.pone.0108080 (PMC4176725; doi:10.1371/journal.pone.0108080)

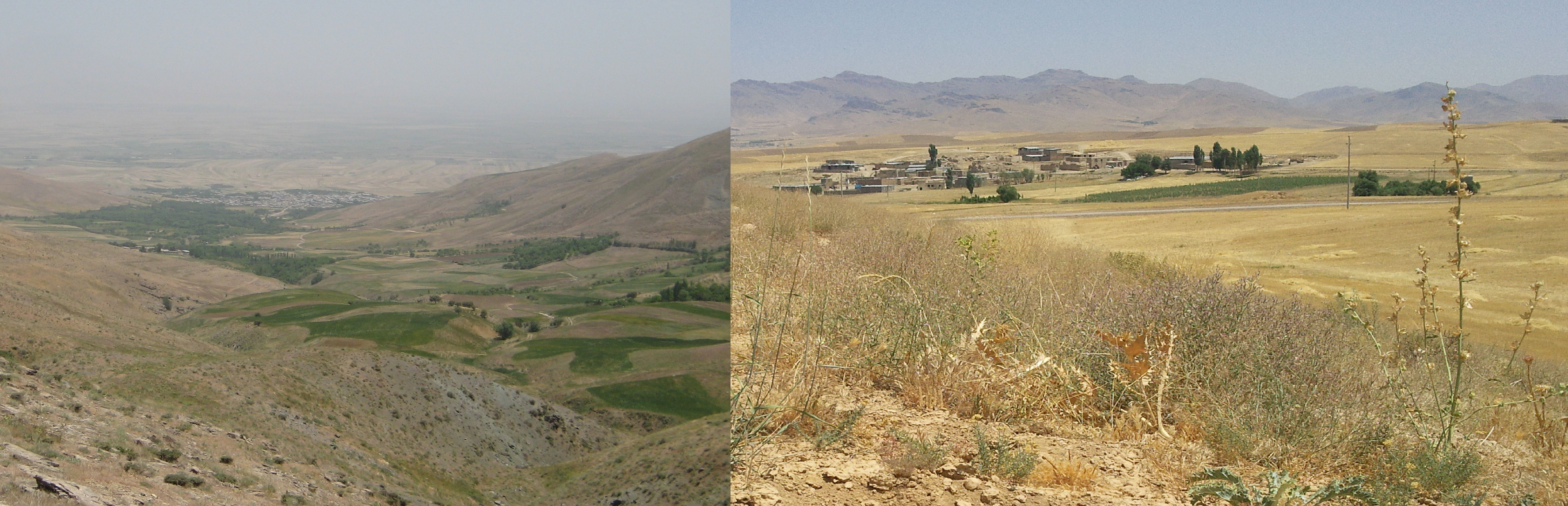

Supplement: Figure S1 — General views of the agroecosystems of Hamedan province, Iran. (JPG) [file pone.0108080.s001.jpg]

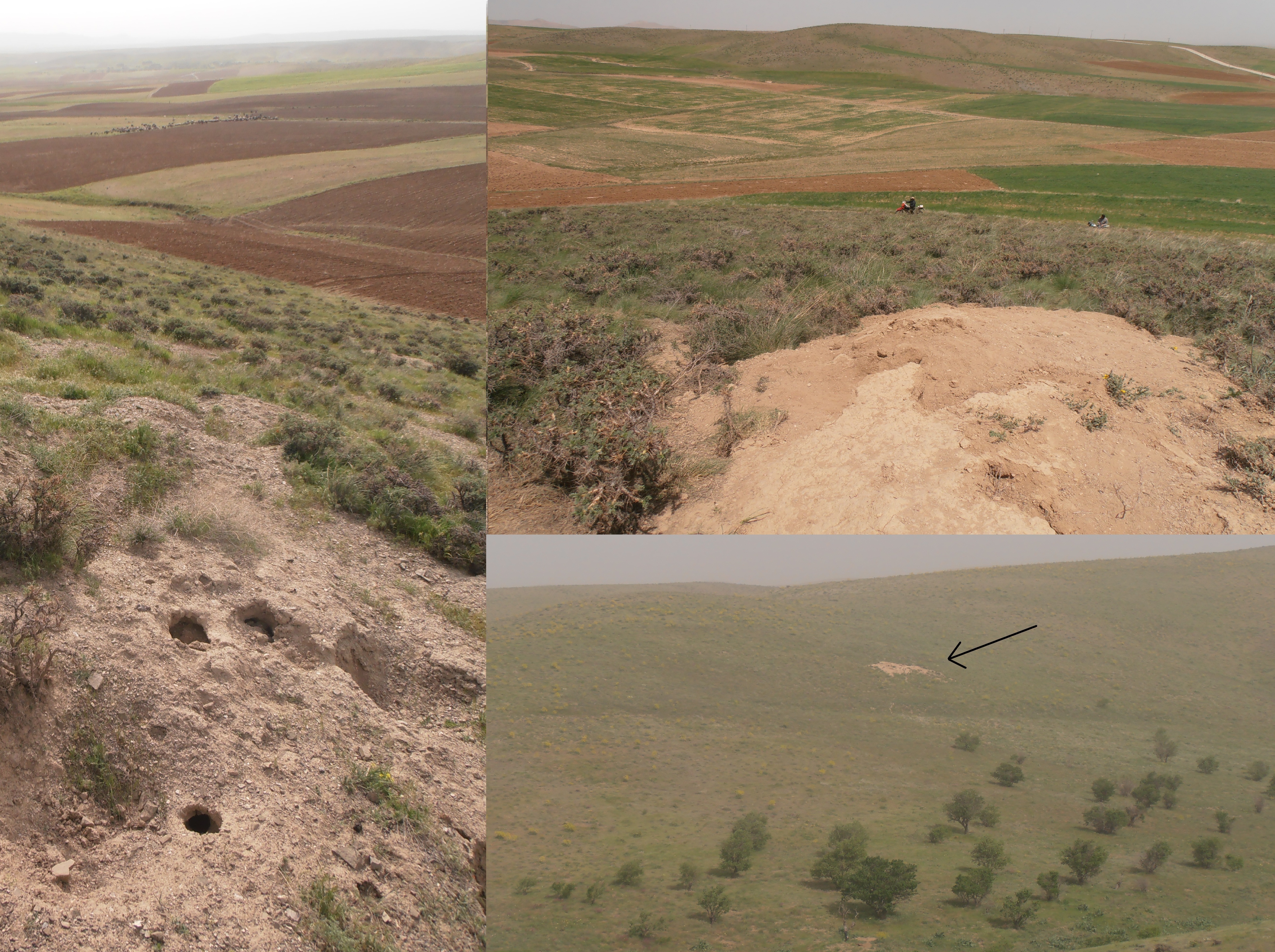

Supplement: Figure S2 — Fine-scale pictures showing the environment around den sites in rangelands of Hamedan province, Iran. (JPG) [file pone.0108080.s002.jpg]
