# Supplementary material for: Spatial Heterogeneity in Human Activities Favors the Persistence of Wolves in Agroecosystems
Source: PLoS One. 2014 Sep 24;9(9):e108080. doi: 10.1371/journal.pone.0108080 (PMC4176725; doi:10.1371/journal.pone.0108080)
Supplement: Table S1 — Results from Generalized Linear Models testing for significant effects of the pairwise interactions between land use and type of roads with elevation and roughness. (DOC) [file pone.0108080.s003.doc]

**Table S1.** Results from Generalized Linear Models testing for significant effects in the pairwise interactions between land uses and type of roads with elevation and roughness. Statistics include Likelihood Ratio (LR) χ^2^ and P-value of Wald χ^2^.

| Models | Terms | LR χ^2^ | *P* of Wald χ^2^ |
| --- | --- | --- | --- |
| Irrigated farms × (Altitude + Roughness) | Irrigated farms | 3.163 | 0.075 |
|  | Altitude | 8.276 | 0.004 |
|  | Roughness | 3.093 | 0.078 |
|  | Irrigated farms × Altitude | 0.461 | 0.496 |
|  | Irrigated farms × Roughness | 6.147 | 0.013 |
| Dry farms × (Altitude + Roughness) | Dry farms | 0.185 | 0.667 |
|  | Altitude | 9.535 | 0.002 |
|  | Roughness | 1.493 | 0.221 |
|  | Dry farms × Altitude | 0.091 | 0.763 |
|  | Dry farms × Roughness | 2.982 | 0.084 |
| Rangeland × (Altitude + Roughness) | Rangeland | 0.701 | 0.402 |
|  | Altitude | 13.988 | 0.001 |
|  | Roughness | 1.011 | 0.314 |
|  | Rangeland × Altitude | 0.797 | 0.371 |
|  | Rangeland × Roughness | 3.725 | 0.087 |
| Range_Dry farms × (Altitude + Roughness) | Range_Dry farms | 9.458 | 0.002 |
|  | Altitude | 7.995 | 0.004 |
|  | Roughness | 2.777 | 0.95 |
|  | Range_Dry farms × Altitude | 1.681 | 0.194 |
|  | Range_Dry farms × Roughness | 0.531 | 0.466 |
| Primary road × (Altitude + Roughness) | Primary road | 6.963 | 0.008 |
|  | Altitude | 7.158 | 0.007 |
|  | Roughness | 3.712 | 0.54 |
|  | Primary road × Altitude | 0.283 | 0.594 |
|  | Primary road × Roughness | 0.159 | 0.689 |
| Secondary road × (Altitude + Roughness) | Secondary road | 0.286 | 0.592 |
|  | Altitude | 12.490 | 0.001 |
|  | Roughness | 1.054 | 0.304 |
|  | Secondary road × Altitude | 3.967 | 0.043 |
|  | Secondary road × Roughness | 1.287 | 0.256 |
