# Supplementary material for: Spatial Heterogeneity in Human Activities Favors the Persistence of Wolves in Agroecosystems
Source: PLoS One. 2014 Sep 24;9(9):e108080. doi: 10.1371/journal.pone.0108080 (PMC4176725; doi:10.1371/journal.pone.0108080)
Supplement: Table S2 — Results of the assessment of goodness-of-fit and discrimination capacity of selected candidate models explaining the selection of den areas/sites by wolves in Hamedan, Iran. (DOC) [file pone.0108080.s004.doc]

**Table S2** Results of the assessment of goodness-of-fit and discrimination capacity of models derived based on GLMs describing den site selection by wolves in Middle-eastern agroecosystem. HL: Hosmer-Lemeshow test to verify goodness-of-fit. Large values of HL χ^2^ and small p-values indicate a lack of fit in the model. Large values for Area Under the ROC Curve (AUC) show excellent discrimination capability of candidate models.

|  | **Log Likelihood** | **HL χ^2^ ( *P*-value)** | **AUC** |
| --- | --- | --- | --- |
| **Fine-scale level** |  |  |  |
| Model 1 | -23.32 | 5.57 (0.69) | 0.916 |
| Model 2 | -22.15 | 3.93 (0.86) | 0.921 |
| Model 3 | -21.43 | 11.47 (0.17) | 0.924 |
| Model 4 | -21.44 | 6.54 (0.58) | 0.933 |
| Model 5 | -22.71 | 5.57 (0.69) | 0.918 |
| Model 6 | -21.55 | 2.12 (0.97) | 0.924 |
| Model 7 | -22.81 | 4.26 (0.83) | 0.916 |
| Model 8 | -23.11 | 7.66 (0.47) | 0.915 |
| **Landscape level** |  |  |  |
| Model 1 | -56.18 | 6.98 (0.54) | 0.818 |
| Model 2 | -59.17 | 6.88 (0.55) | 0.825 |
| Model 3 | -55.95 | 4.44 (0.81) | 0.822 |
| Model 4 | -56.16 | 10.24 (0.25) | 0.823 |
| Model 5 | -59.33 | 7.55 (0.65) | 0.822 |
| Model 6 | -54.83 | 4.49 (0.59) | 0.761 |
| Model 7 | -55.08 | 4.44 (0.82) | 0.821 |
